# Case Report: Gastric submucosal neoplasm with *CTNNB1* mutation showing *GLI1* overexpression and epithelial differentiation

**DOI:** 10.3389/fmed.2025.1526614

**Published:** 2025-04-03

**Authors:** Karin Ashizawa, Tsuyoshi Saito, Yukinori Yube, Shinji Mine, Tetsu Fukunaga, Cristina R. Antonescu, Takashi Yao

**Affiliations:** ^1^Department of Human Pathology, Graduate School of Medicine, Juntendo University, Bunkyo-ku, Japan; ^2^Intractable Disease Research Center, Graduate School of Medicine, Juntendo University, Bunkyo-ku, Japan; ^3^Department of Upper Gastroenterological Surgery, Juntendo University Hospital, Bunkyo-ku, Japan; ^4^Department of Pathology, Memorial Sloan Kettering Cancer Center, New York, NY, United States

**Keywords:** pseudoendocrine sarcoma, stomach, *CTNNB1*, cytokeratin, epithelial, neuroendocrine, *GLI1*

## Abstract

New disease entities have been emerging based on molecular pathological findings, such as pseudoendocrine sarcoma and mesenchymal neoplasm with *GLI1* gene alterations, which resemble well-differentiated neuroendocrine tumors. We report a unique case of a gastric submucosal neoplasm of approximately 1.5 cm in size with *CTNNB1* mutation showing *GLI1* overexpression and epithelial differentiation in a 66-year-old man. It was incidentally identified by routine health screening, and was a slowly growing tumor. Macroscopically, it was a slightly protruded tumor into the mucosa, and was primarily located from the submucosa to the muscularis propria. It was a well-defined lesion measured approximately 20 mm, and was almost stable during almost 5 years after initial identification of the tumor. Uniform round-to-epithelioid cells arranged in solid trabeculae with a microtubular/acinar appearance were seen microscopically. Occasional mitotic figures were noted, but no necrosis was observed. Immunohistochemistry (IHC) demonstrated diffuse expression of pan-cytokeratin, CD10, and CD56 without neuroendocrine markers (chromogranin A, synaptophysin, and INSM1). Molecular analysis confirmed the presence of a hot spot *CTNNB1* mutation (S33C), supported by diffuse *β*-catenin nuclear expression by IHC. Further molecular investigations revealed the absence of *GLI1* gene rearrangements, *GLI1* amplification, and other fusions. Several differential diagnoses were considered; however, none adequately fit the criteria. The patient remained disease-free for 24 months postoperatively without further adjuvant therapy.

## Introduction

Molecular pathological research is rapidly advancing, and new disease entities that are based on molecular pathological findings have emerged. For example, disease entities that resemble well-differentiated neuroendocrine tumors, such as pseudoendocrine sarcoma, mesenchymal neoplasms with *GLI1* gene alterations described as “distinctive nested glomoid neoplasm,” gastroblastoma, and plexiform fibromyxoma have been reported (1–7). Gastroblastoma is a distinctive biphasic stomach tumor that shows epithelioid or spindle cells and harbors the *MALAT::GLI1* fusion gene (5). Pseudoendocrine sarcoma is a recently recognized entity among soft tissue sarcomas, characterized by its close resemblance to well-differentiated neuroendocrine tumors, but lacking epithelial/neuroendocrine differentiation by immunohistochemistry (IHC). Additionally, nearly all cases exhibit aberrant nuclear accumulation of *β*-catenin caused by the secondary activating hot spot *CTNNB1* mutations (1). These lesions commonly occur in deep soft tissues (1–4). These entities are morphologically similar to well-differentiated neuroendocrine tumors, which often makes their diagnosis difficult. Herein, we report a unique case of a gastric submucosal tumor that demonstrated strong expression of monoclonal antibodies directed against keratins in the absence of neuroendocrine differentiation by IHC. However, this case did not fit any of these criteria, and we report it as a gastric submucosal neoplasm with *CTNNB1* mutation, showing *GLI1* overexpression and epithelial differentiation.

## Case presentation

### Clinical course

In 2017, a routine health screen incidentally revealed a submucosal gastric tumor in a 66-year-old man. The tumor located on the lesser curvature of the cardia, measuring approximately 1.5 cm, has been monitored since its discovery. Endoscopic ultrasonography (EUS) showed an almost uniform hypoechoic area contiguous to the fourth layer. There was no obvious non-echoic area or blood flow signal. While initially clinically diagnosed as leiomyoma, the possibility of a gastrointestinal stromal tumor was considered. EUS-guided fine needle aspiration (EUS-FNA) in May 2022 revealed a tumor characterized by the monotonous proliferation of epithelioid cells. IHC analysis demonstrated positive staining for CD56 but negative staining for chromogranin A, synaptophysin, and INSM-1. The MIB-1-labeling index (LI) was approximately 2%, which led to the diagnosis of a grade 1 (G1) neuroendocrine tumor, although the immunohistochemical findings were inconsistent. Upon admission, laboratory testing revealed slight abnormalities in renal function. The tumor was expected by EUS to be located in the submucosa and deeper, so the endoscopic resection was considered to be difficult. Therefore, proximal gastrectomy and lymph node dissection was performed by robotic technique in June 2022 as part of the patient’s management. The tumor was located on the lesser curvature of the cardia, allowing resection without confirmation of the tumor location during surgery, despite its small size. The operative time was 5 h and 5 min, with a blood loss of 10 mL. On 10 days after surgery, the patient developed a fever. Gastroendoscopy was performed and it revealed leakage on the left side of the esophageal margin slightly apart from the anastomotic site. Stomach tube was placed for drainage, and a central venous catheter (CV) was inserted. At 1 month after surgery, leakage improved and the drain tube was removed. However, the patient developed a fungal infection and fungal endophthalmitis associated with CV insertion. The patient received intravenous antifungal treatment for additional 1 month, then changed to oral medication. Finally, the patient discharged 2 months post-surgery.

### Pathological findings of the surgically resected specimen

**Figure 1:**
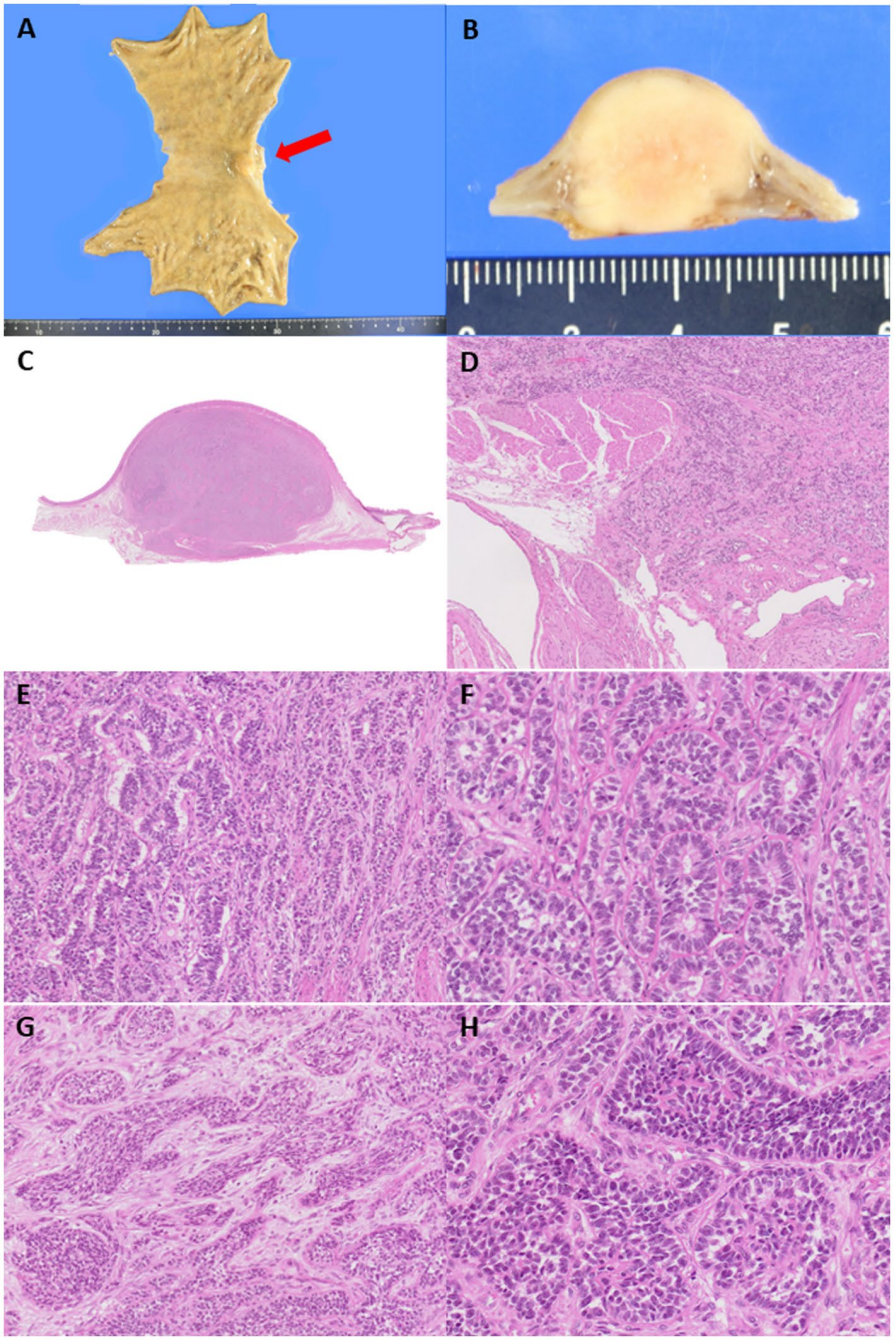
**(A)** A submucosal-centered tumor with slight protrusion toward the mucosal aspect is identified at the cardia and lesser curvature (red arrow). **(B)** The cut surface of the tumor shows a well-demarcated, white-yellow solid lesion measuring 20 mm in the largest dimension. **(C)** The tumor extends from the submucosa to the muscularis propria. **(D,E)** Tumor cells are arranged in microacinar and trabecular architectural arrangements without necrosis, reminiscent of a well-differentiated neuroendocrine tumor. The tumor mainly appeared well-demarcated but irregularly infiltrated the surrounding tissue in a few areas. **(F)** Tumor cells are monomorphic and exhibited scant, pale eosinophilic cytoplasm, round nuclei, and speckled chromatin. Occasional rosette-like structural formations are observed, similar to those observed in well-differentiated neuroendocrine tumors. **(G)** In a few areas, short, spindle-shaped cells arranged in tight nests are observed within the fibromyxoid stroma. **(H)** Tumor cells form solid sheets with small amounts of hyalinized stromal bodies.

Macroscopically, the tumor exhibited a slight protrusion into the mucosa (Figure 1) but was primarily located from the submucosa to the muscularis propria. This well-defined lesion measured approximately 20 mm. Microscopically, the tumor was composed of uniform round-to-epithelioid cells arranged in solid trabeculae with a microtubular/acinar appearance. Tumor cells showed scant eosinophilic cytoplasm and monomorphic round-to-ovoid nuclei with fine chromatin and no pleomorphism. Occasional mitotic figures were observed (1/50 high power fields) and no necrosis was observed. In a few areas, short spindle-shaped cells arranged in tight nests were noted within the fibromyxoid stromal background (Figure 1). Totally, 31 lymph nodes were retrieved and no metastases were identified.

**Figure 2:**
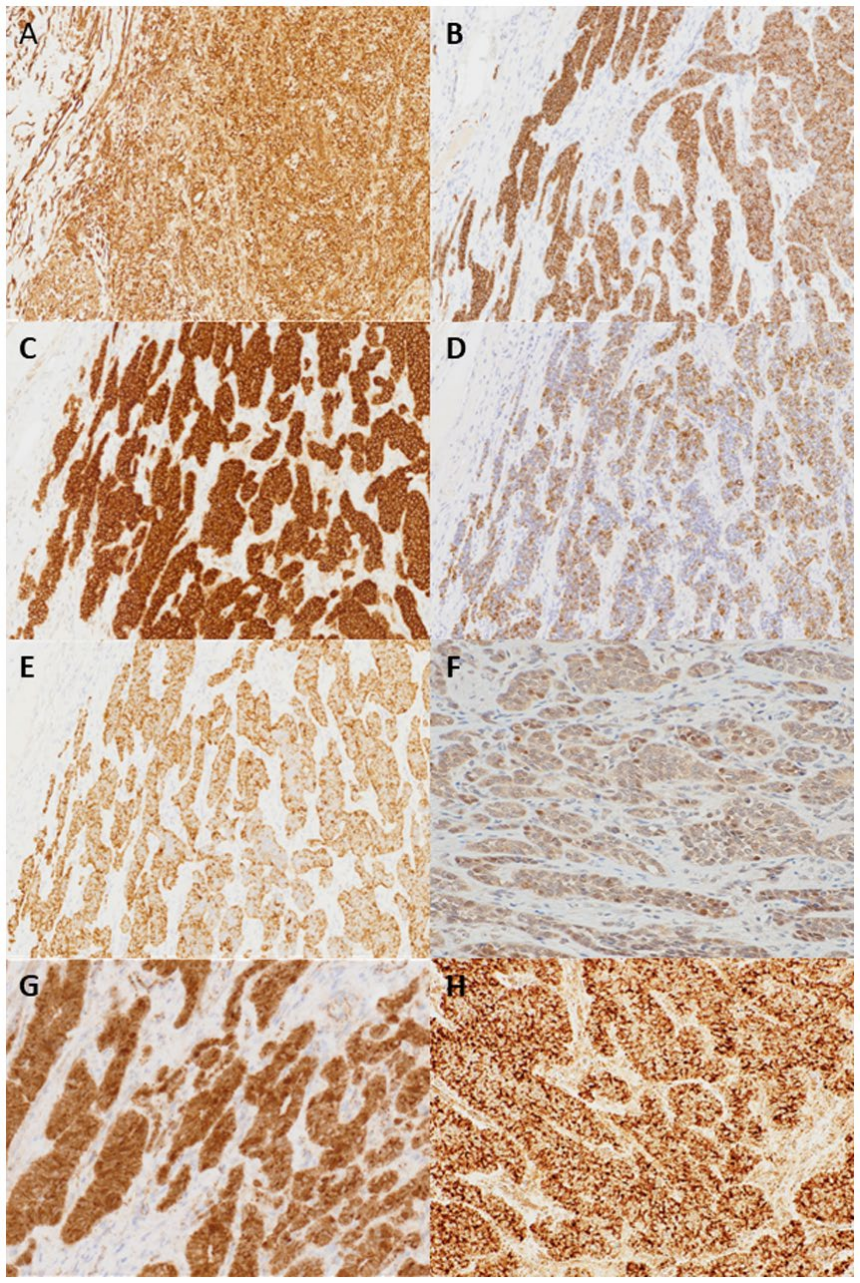
Tumor cells are positive for vimentin **(A)**, CD56 **(B)**, CD10 **(C)**, CAM5.2 **(D)**, AE1/3 **(E)**. Focal and weak nuclear staining for *GLI1* **(F)** and cytoplasmic and partial nuclear staining for *β*-catenin **(G)** are also noted. STAT6 IHC shows cytoplasmic staining but not nuclear staining **(H)** (×200).

IHC analysis revealed that the tumor cells were diffusely positive for vimentin, CD56, CD10, CAM5.2, AE1/AE3, estrogen receptor (ER), progesterone receptor (PgR) and androgen receptor (AR), STAT6 (cytoplasmic), and focally positive for S-100, E-cadherin, cyclinD1 and ATRX, whereas they were negative for chromogranin A, synaptophysin, INSM-1, smooth muscle antibody, c-kit, calretinin, epithelial membrane antigen, inhibin, SALL4, AFP, Glypican-3, p53, p40, CD34, bcl-10, SF-1, PAX-8, CDX2, CD99, MelanA, CEA, CA19-9, and CA125 (Figure 2). As this immune profile was deemed nondiagnostic for neuroendocrine neoplasms, further molecular studies were performed. As one of the primary differential diagnoses was gastroblastoma, RT-PCR for *MALAT1::GLI1* fusion was performed, which yielded a negative result. Furthermore, *DDIT3/GLI1* FISH analysis was negative for *GLI1* gene rearrangements, and targeted RNA sequencing using the Illumina TruSight panel (500 genes) was negative for gene fusion candidates. RNA sequencing was predominantly performed to identify gene fusions rather than mutations, and coincidentally, manual inspection revealed a 10-fold increase in the mRNA expression of *STAT6* and *GLI1*; however, no gene amplification was detected by FISH. Additional IHC showed focal and weak staining for *GLI1* of uncertain significance.

**Figure 3:**
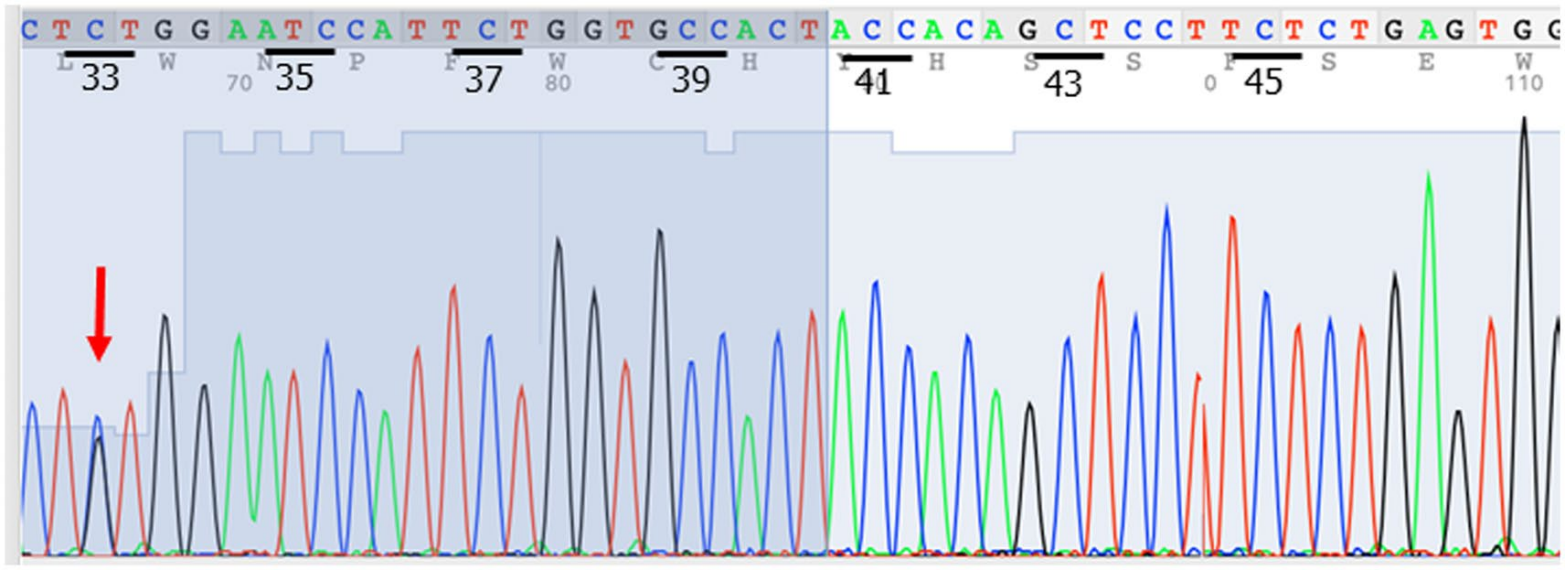
Sanger sequencing confirmed the *CTNNB1* mutation (S33C) (red arrow).

The next possibility to be considered was a pseudoendocrine sarcoma. *β*-catenin immunostaining was performed, which showed aberrant nuclear expression (Figure 2). This result was further confirmed by Sanger sequencing, which showed a hot spot *CTNNB1* mutation (S33C) (Figure 3). Consistent with these results was the tumor content of 80% in this case, which equated to an allele frequency of the *CTNNB1* mutation (S33C) of 40%. Therefore, the *CTNNB1* mutation was deemed to be clonal.

## Discussion

Gastric mesenchymal tumors are rare, with gastrointestinal stromal tumors, schwannomas, and leiomyomas being the most common. Based on the histological features observed in the initial biopsy, a diagnosis of a well-differentiated neuroendocrine tumor was considered; however, the IHC findings were not fully supportive. Gastric neuroendocrine neoplasms typically exhibit positive immunohistochemical staining for chromogranin A, synaptophysin, and INSM-1. In contrast, IHC findings suggested the possibility of gastroblastoma (5). However, gastroblastomas, similar to synovial sarcomas, usually exhibit a biphasic pattern characterized by a mixture of spindle cells and nests of epithelioid cells with abundant eosinophilic cytoplasm. These features were not observed in the present case. In addition, RT-PCR, FISH, and RNA sequencing failed to identify the presence of *GLI1* gene rearrangements, *MALAT1::GLI1* fusion, *PTCH1::GLI1* fusion, or an *EWSR1-CTBP1*, recently identified in a patient with Wiskott-Aldrich syndrome (6, 8). Therefore, a diagnosis of gastroblastoma was excluded. The next possibility was a pseudoendocrine sarcoma. *CTNNB1* mutation and nuclear expression for *β*-catenin IHC are consistent with pseudoendocrine sarcoma; however, these sarcomas occur commonly in the truncal locations such as the soft tissues of the vertebral body region and posterior head (1), so the site, in this case, did not fit pseudoendocrine sarcoma. In addition, short spindle cells arranged in tight nests were observed within a fibromyxoid stromal background that did not fit pseudoendocrine sarcoma. The case also showed focal and weak staining expression of *GLI1* on IHC and monoclonal antibodies directed against keratins, such as CAM5.2 and AE1/AE3, although prior studies have not reported any positive staining for monoclonal antibodies directed against keratins and *GLI1* on IHC (1, 2, 9). Based on these findings, the diagnosis of pseudoendocrine sarcoma was excluded.

The presence of the *CTNNB1* mutation and the morphological similarity in this case may raise the possibility of a solid pseudopapillary neoplasm or Sertoli-like neoplasm, such as a Sertoli cell tumor of the testis. However, ectopic pancreatic tissue was not observed in the present case. Furthermore, the absence of a history of gonadal systemic tumors and no abnormalities in the gonads on PET-CT ruled out this possibility. However, the overexpression of hormone receptors such as ER, PgR, and AR in this case remains puzzling.

The possibility of a mesenchymal neoplasm with *GLI1* gene alterations was raised because of *GLI1* mRNA and protein overexpression (7). Recent studies have highlighted the morphological similarities between well-differentiated neuroendocrine tumors and soft tissue sarcomas with *GLI1* gene alterations described as “distinctive nested glomoid neoplasm” (1, 7, 10, 11). However, it was excluded because of the absence of *GLI1* fusion and the presence of the *CTNNB1* mutation. Furthermore, irregular fibrous septa, lobular growth patterns, a prominent capillary network, or lobules protruding into vascular lumina beneath intact endothelium are frequently seen in distinctive nested glomoid neoplasms, but were not observed in this case. A study by Parrack et al. reported that GLI1 IHC was highly sensitive (91.3%) and specific (98.0%) for mesenchymal tumors with driver *GLI1* alterations among morphological mimics (9). However, in this case, GLI1 IHC was focally and weakly positive rather than diffusely and strongly positive, which does not positively support a GLI1-altered mesenchymal neoplasm.

Furthermore, the strong and diffuse cytokeratin expression in this case seemed unusual for a *GLI1*-altered mesenchymal neoplasm (12). Since this case does not fit the disease concepts reported to date, we report it as “gastric submucosal neoplasm with *CTNNB1* mutation showing *GLI1* overexpression and epithelial differentiation.”

Regarding the prognosis of this case, the patient was followed up for almost 5 years without any treatment before undergoing surgery, during which the tumor size remained relatively stable. The MIB-1 proliferation index in the surgical specimen was approximately 5%, and the patient survived for 2 years without any evidence of recurrence or metastasis after surgery. Gastroendoscopy has been performed every year after surgery, and no new lesions are observed. These outcomes show the slow growth and low-grade nature of this tumor. This case report provides new insights into the clinicopathological characteristics of this disease.

## Data Availability

The original contributions presented in the study are included in the article/supplementary material, further inquiries can be directed to the corresponding author/s.

## References

[1] Papke DJ Jr Dickson BC Sholl L Fletcher CDM. Pseudoendocrine sarcoma: Clinicopathologic analysis of 23 cases of a distinctive soft tissue neoplasm with metastatic potential, recurrent CTNNB1 mutations, and a predilection for truncal locations. Am J Surg Pathol. (2022) 46:33–43. doi: 10.1097/PAS.0000000000001751, PMID: 34081037

[2] Vizcaino MA Folpe AL Huffman H Panchal RR Nielsen GP Kipp BR. Pseudoendocrine sarcoma: clinicopathologic, molecular, and epigenetic features of one case. Virchows Arch. (2023) 483:899–904. doi: 10.1007/s00428-023-03695-3, PMID: 37953374

[3] Bellan E Zanco F Baciorri F Toffolatti L Dei Tos AP Sbaraglia M. Case report: pseudoendocrine sarcoma, a clinicopathologic report of a newly described soft tissue neoplasm. Virchows Arch. (2023) 482:1057–63. doi: 10.1007/s00428-022-03476-4, PMID: 36564514

[4] Moran JMT Hung YP Selig MK Nielsen GP. Meningioma-like ultrastructural features of Pseudoendocrine sarcoma. Am J Surg Pathol. (2022) 46:1014–6. doi: 10.1097/pas.0000000000001890, PMID: 35297787

[5] Graham RP Nair AA Davila JI Jin L Jen J Sukov WR. Gastroblastoma harbors a recurrent somatic MALAT1-GLI1 fusion gene. Mod Pathol. (2017) 30:1443–52. doi: 10.1038/modpathol.2017.68, PMID: 28731043

[6] Koo SC LaHaye S Kovari BP Schieffer KM Ranalli MA Aldrink JH. Gastroblastoma with a novel EWSR1-CTBP1 fusion presenting in adolescence. Genes Chromosomes Cancer. (2021) 60:640–6. doi: 10.1002/gcc.22973, PMID: 34041825

[7] Papke DJ Jr Dickson BC Oliveira AM. Distinctive nested Glomoid neoplasm: Clinicopathologic analysis of 20 cases of a mesenchymal neoplasm with frequent GLI1 alterations and indolent behavior. Am J Surg Pathol. (2023) 47:12–24. doi: 10.1097/pas.0000000000001979, PMID: 36395474

[8] Chen C Lu J Wu H. Case report: submucosal gastroblastoma with a novel PTCH1::GLI2 gene fusion in a 58-year-old man. Front Oncol. (2022) 12:935914. doi: 10.3389/fonc.2022.935914, PMID: 36147912 PMC9487307

[9] Parrack PH Mariño-Enríquez A Fletcher CDM Hornick JL Papke DJ. GLI1 immunohistochemistry distinguishes mesenchymal neoplasms with GLI1 alterations from morphologic mimics. Am J Surg Pathol. (2023) 47:453–60. doi: 10.1097/pas.0000000000002018, PMID: 36693363

[10] Antonescu CR Agaram NP Sung YS Zhang L Swanson D Dickson BC. A distinct malignant epithelioid neoplasm with GLI1 gene rearrangements, frequent S100 protein expression, and metastatic potential: expanding the Spectrum of pathologic entities with ACTB/MALAT1/PTCH1-GLI1 fusions. Am J Surg Pathol. (2018) 42:553–60. doi: 10.1097/pas.0000000000001010, PMID: 29309307 PMC5844813

[11] Agaram NP Zhang L Sung YS Singer S Stevens T Prieto-Granada CN. GLI1-amplifications expand the spectrum of soft tissue neoplasms defined by GLI1 gene fusions. Mod Pathol. (2019) 32:1617–26. doi: 10.1038/s41379-019-0293-x, PMID: 31189998 PMC6821565

[12] Saoud C Agaimy A Dermawan JK Chen JF Rosenblum MK Dickson BC. A comprehensive Clinicopathologic and molecular reappraisal of GLI1-altered mesenchymal tumors with pooled outcome analysis showing poor survival in GLI1- amplified versus GLI1-rearranged tumors. Am J Surg Pathol. (2024) 48:1302–17. doi: 10.1097/pas.0000000000002272, PMID: 38934567 PMC12150273

